# Analysis of Wheat Pollen Ole E I Proteins Reveals Potential Roles in Fertility and Stress Adaptation

**DOI:** 10.3390/ijms26167707

**Published:** 2025-08-09

**Authors:** Jinghong Zuo, Yanfeng Jia, Weiwei Wang, Chunman Guo, Zhaofeng Fang, Yujuan Zhang, Jinzhou Fu, Sijia Zhao, Changping Zhao, Dezhou Wang, Guohang Yang, Yimiao Tang

**Affiliations:** 1 Institute of Hybrid Wheat, Beijing Academy of Agriculture and Forestry Sciences, Beijing 100097, China; zuojinghong@baafs.net.cn (J.Z.); ruchu9652@foxmail.com (Y.J.); wangweiwei@baafs.net.cn (W.W.); guochunman@baafs.net.cn (C.G.); fang-zhaofeng-hi@163.com (Z.F.); zhangyujuan125@163.com (Y.Z.); fu_jinzhou@163.com (J.F.); 17737863405@163.com (S.Z.); zhaochangping@baafs.net.cn (C.Z.); wangdezhou84@126.com (D.W.); 2 The Municipal Key Laboratory of the Molecular Genetics of Hybrid Wheat, Beijing 100097, China; 3 Agriculture College, Yangtze University, Jingzhou 434023, China

**Keywords:** abiotic stresses, anther development, expression pattern, Pollen *Olea europaea* I (POEI), thermo-sensitive genic male sterile

## Abstract

Abiotic stresses increasingly threaten wheat (*Triticum aestivum* L.) productivity by impairing pollen development and fertilization, yet the molecular regulators that coordinate reproductive success with environmental resilience remain underexplored. Here, we present a comprehensive genome-wide analysis of the Pollen *Olea europaea* I (POEI) protein family in common wheat. A total of 104 *TaPOEI* genes were identified and classified into six phylogenetic clades, each sharing conserved exon–intron structures and key protein motifs. Promoter analysis revealed abundant cis-elements associated with phytohormone signaling and abiotic stress responses. Notably, *TaPOEI 16-A* was preferentially expressed in anthers, showing high expression during early anther development and responding to both high- and low-temperature stresses. Pairwise comparison between thermosensitive genic male-sterile wheat lines and fertile lines suggests a potential role for *TaPOEI 16-A* in regulating male fertility in response to temperature fluctuations. Our comprehensive analysis establishes a foundation for future functional studies of the *TaPOEI* family and provides insights into wheat fertility and stress resilience enhancement.

## 1. Introduction

Wheat (*Triticum aestivum* L.) is one of the world’s most important staple cereals, supplying nearly 20% of the calories and proteins consumed by humans [1]. However, its productivity is increasingly threatened by the rising frequency and intensity of abiotic stresses under climate change [1,2]. Since pollen development and fertilization are highly sensitive to environmental fluctuations, disruptions in these reproductive processes can directly compromise yield and grain quality, posing serious challenges to global food security [3,4,5].

The Pollen *Olea europaea* I (POEI) protein family, initially identified through the olive pollen allergen Ole e I, comprises a group of small, secreted proteins capable of triggering IgE-mediated allergic reactions in humans [6,7,8,9,10]. Beyond their allergenic properties, POEI proteins play essential biological roles in plants, particularly in pollen development processes such as hydration, germination, and tube elongation [3,4,5]. The characteristic POEI domain is conserved across more than 100 plant species and is catalogued in the Pfam database as the “pollen protein of the Ole e 1 family” [11,12]. Typical Ole e 1 proteins consist of a single-chain peptide of 145 amino acids and contain six cysteine residues that facilitate disulfide bond formation, along with the conserved sequence [EQT]-G-X-V-Y-C-D-[TNP]-C-R (where X represents any residue), which likely underlies *POEI* gene evolution [13,14,15].

Genomic studies have revealed the widespread distribution and functional diversification of *POEI* genes across species. For example, 28 *POEI* genes have been identified in *Arabidopsis thaliana*, 45 in *Oryza sativa*, 45 in *Pyrus bretschneideri*, and a drought-inducible member (*GmPOI*) in soybean (*Glycine max*) [14,16,17,18]. In rice, eight of the 45 *POEI* genes exhibit spike-specific expression [16], while in pear, five exhibit pollen-preferential expression [14], suggesting roles in reproductive development. Nevertheless, most *POEI* family members are expressed across various tissues, including roots, leaves, and spikes, which indicates potential roles in specific developmental or physiological stages.

Functional evidence from various species supports the involvement of POEI proteins in both fertility and stress responses. In olive, Ole e I localizes to the tapetum and outer wall of pollen grains, where it is essential for proper pollen tube dynamics [4,6]. Homologous proteins in other species—such as *LAT52* and *Pollen extensin-like* (*tPex*) in tomato (*Solanum lycopersicum*) and *mPex2* in maize (*Zea mays*)—are similarly required for pollen development and structural integrity [19,20,21,22]. In pepper (*Capsicum annuum*), *CaOle e 6* is differentially expressed in male-sterile and fertile strains, showing higher expression during late anther microspore development in fertile strains [23]. In soybean, the *GmPO I* gene is transcriptionally induced by abiotic stressors and phytohormones, and its overexpression enhances drought tolerance [17]. In *Arabidopsis thaliana*, *AtSAH7*—an Ole e I domain-containing protein—interacts with the selenium-tolerance protein *AtSBP1* and participates in the reactive oxygen species (ROS)-mediated stress response [24]. Likewise, the pear *PbrPOE21* inhibits pollen tube growth in vitro by modulating apical ROS levels, further supporting a conserved role for POEI proteins in coordinating pollen development with oxidative stress signaling [14].

Collectively, *POEI* genes form a highly conserved superfamily with critical roles in both reproductive development and environmental adaptation. Yet, a comprehensive analysis of *POEI* genes in wheat—an allohexaploid with a complex genome—remains lacking. In this study, we present a comprehensive analysis of *POEI* genes within the wheat genome, identifying 104 genes encoding proteins with the conserved POEI domain, and elucidating their phylogenetic relationships and evolutionary trajectories. We further investigated their tissue-specific expression and stress responsiveness under drought, salinity, cold, and heat conditions. Notably, several *TaPOEI* genes exhibited pollen-preferential expression, and members such as *TaPOEI 16-A* were responsive to temperature fluctuations in thermosensitive genic male-sterile (TGMS) lines, highlighting their potential role in fertility regulation. These findings provide valuable insights into the molecular mechanisms underlying wheat reproduction and stress adaptation, and establish a foundation for future functional studies and hybrid breeding strategies.

## 2. Results

### 2.1. Genome-Wide Identification and Chromosomal Distribution, and Evolutionary Analysis of Putative POEI Genes in Wheat: Insights into Environmental Adaptation

In this study, we performed a genome-wide identification of the *POEI* gene family in wheat. As queries, we used 28 experimentally characterized POEI proteins from *Arabidopsis thaliana* and an expanded set of 52 POEI sequences from rice (sourced from https://www.ricedata.cn/gene (accessed on 6 January 2024)). These reference sequences served as inputs for BLASTP and HMMER searches against the IWGSC RefSeq v2.1 wheat proteome. Candidate proteins were further validated by confirming the presence of the conserved POEI domain via the Pfam and NCBI Conserved Domain Databases, resulting in the identification of 104 non-redundant POEI homologs in wheat (Table S1). These 104 putative *TaPOEI* genes are distributed across 18 of the 21 wheat chromosomes, with no *TaPOEI* genes present on chromosomes 7A, 7B, or 7D (Figure S1). The genes were named based on the homologous correspondence with *OsPOEI* genes in the rice genome, as well as their chromosomal locations and phylogenetic relationships. Among the three subgenomes, the A subgenome contains 34 *TaPOEI* genes, while the B and D subgenomes harbor 31 and 27 genes, respectively. Chromosomes 1 to 6 exhibit varied gene densities, with the highest concentrations of *TaPOEI* genes on chromosomes 2 (28% of total) and 3 (22% of total). Specifically, chromosome 2A contains the highest number of *TaPOEI* genes, with 14 identified members, followed by chromosome 2B, which holds 9 genes. Most chromosomes contain between three and five *TaPOEI* genes, with the exception of chromosome 5, with each homoeolog harboring two genes. The identified TaPOEI proteins vary significantly in length, ranging from 99 to 522 amino acids (aa), with TaPOEI 4-D being the longest (522 aa). The predicted molecular weights of these proteins range from 10.25 kDa (TaPOEI 15c-U.3) to 52.74 kDa (TaPOEI 4-D). Additionally, the isoelectric points (pI) of these proteins also show considerable variation, from a low of 4.84 (TaPOEI 4-D) to a high of 11.13 (TaPOEI 29-B), with most proteins exhibiting pI values above 7, indicating their hydrophilic nature (Table S1).

**Figure 1:**
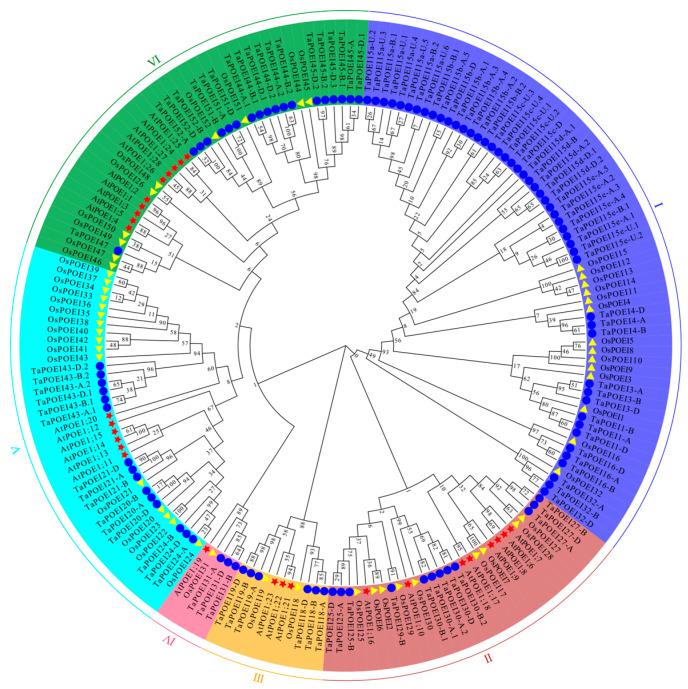
Phylogenetic analysis of POEI proteins. A maximum likelihood phylogenetic tree was reconstructed using POEI proteins from *Arabidopsis thaliana* (At, indicated by red stars), *Oryza sativa* (Os, indicated by yellow triangles), and *Triticum aestivum* (Ta, indicated by blue circles) with MEGA-X software. The tree illustrates the evolutionary relationships among the POEI protein family members across both species.

To investigate the evolutionary relationships of the POEI family and predict its classification in wheat, we constructed a phylogenetic tree using conserved protein sequences from 184 proteins, including 28 from Arabidopsis, 52 from rice, and 104 from wheat. The maximum-likelihood method was applied using MEGA software (version 10.1.7) to build the phylogenetic tree. Based on the clustering of the phylogenetic tree, genes with closer evolutionary relationships were grouped together, resulting in six distinct groups within the POEI family (Figure 1). This classification largely aligns with the phylogenetic grouping of Arabidopsis POEI proteins, thereby supporting a conserved evolutionary trajectory across species [18]. Group I contains the largest number, including 49 wheat *POEIs* and 14 rice *POEIs*, but no Arabidopsis *POEI* genes. This group is exclusively composed of wheat POEI proteins, encoded by genes located on chromosome 2, suggesting a potential chromosomal hotspot for TaPOEI protein evolution. Group II represents eight Arabidopsis proteins (AtPOE1; 6–10 and AtPOE1; 16–18), 12 wheat genes, and 9 rice proteins. Group III includes two rice proteins (OsPOEI 18 and OsPOEI 19), three Arabidopsis POEIs, and six wheat POEIs mapping to chromosomes 2 and 5. Group IV consists of a single rice protein (OsPOEI 31), one Arabidopsis protein (AtPOE1;19), and three wheat genes located on chromosome 1. Group V includes six Arabidopsis POEIs, 16 rice POEIs (OsPOEI 20–23, OsPOEI 33–43), and 15 wheat POEIs. Notably, AtPOE1;20 (also reported as PROLINE-RICH PROTEIN-LIKE 1 [*AtPRPL1*]), which has been implicated in the salinity stress response in Arabidopsis roots [17,18], is phylogenetically related to the wheat proteins *TaPOEI 43-A.1*, *TaPOEI 43-B.1*, and *TaPOEI 43-D.1*. Lastly, Group VI contains 10 Arabidopsis and 10 rice proteins, along with 19 wheat proteins, with the wheat proteins being predominantly encoded by genes mapping to chromosomes 1 and 3. This phylogenetic analysis not only provides insight into the evolutionary history of the *POEI* gene family in wheat but also highlights specific gene clusters that may play critical roles in wheat acclimation to environmental stresses.

### 2.2. Structural and Regulatory Features of TaPOEI Genes: Insights from Motif and Promoter Analysis

We conducted a detailed analysis of the conserved motifs in TaPOEI proteins using the MEME online tool, identifying 10 conserved motifs, designated motifs 1 to 10 (Figure S2). The phylogenetic analysis grouped the TaPOEI family members into three distinct clades, designated Groups I to III. Among these, motifs 1, 6, and 9 were the most conserved, appearing in most TaPOEI proteins across all groups. Proteins from Groups I and III had more conserved motifs than those in Group II. Specifically, all TaPOEI proteins in Group I contained motifs 6, 8, and 9, while Group II was characterized by the presence of motifs 1, 4, and 5. Motifs 1, 3, 4, and 7 were unique to Group III, suggesting that members within each group share conserved domains that may be associated with their specific biological functions.

An analysis of gene structure revealed that the *TaPOEI* family members exhibit consistent structural patterns across different phylogenetic branches (Figure S2). Within each group, genes generally share similar exon–intron configurations, with most possessing one intron and two exons. This structural conservation within groups aligns with the high similarity observed in the conserved motifs present in their encoded proteins and supports their phylogenetic classification.

Furthermore, an analysis of promoter cis-regulatory elements within the *TaPOEI* gene family uncovered cis-acting elements with potential regulatory functions. These elements were categorized into three main types based on their predicted roles: phytohormone-responsive, defense- and stress-responsive, and growth and development regulatory elements (Figures S2 and S3). Among the phytohormone-responsive elements identified were those responsive to abscisic acid, methyl jasmonate, gibberellin, auxin, and salicylic acid. Additionally, we detected several abiotic stress-related cis-acting elements, including multiple low-temperature-responsive elements and MYB-binding sites associated with drought inducibility. These findings suggest that the *TaPOEI* genes are regulated by a complex network of promoter elements that contribute to their functional diversity under various physiological and environmental conditions.

### 2.3. Interactions Between miRNAs and TaPOEI Genes: Implications for Wheat Reproductive Development

Plant microRNAs (miRNAs), non-coding small RNAs typically 21-24 nucleotides in length, play a crucial role in regulating gene expression, particularly during reproductive development [25,26]. Through an analysis of existing small RNA databases, we identified 14 miRNAs that are potentially involved in the regulation of the *TaPOEI* gene family based on their sequence complementarity: tae-miR1120a, tae-miR1124, tae-miR1129, tae-miR397-5p, tae-miR5086, tae-miR531, tae-miR5384-3p, tae-miR9657a-3p, tae-miR9661-5p, tae-miR9669-5p, tae-miR9676-5p, tae-miR9677a, tae-miR9773, and tae-miR9780.

**Figure 2:**
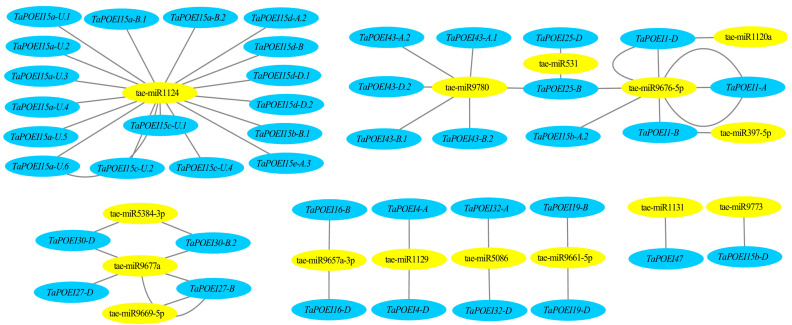
Diagram of the regulatory networks between miRNAs and their putative target *TaPOEI* genes. miRNAs are indicated by yellow coloring, while genes are represented in blue.

Among these, tae-miR1124, previously reported to be involved in regulating anther development in male-sterile wheat, was predicted to target 18 *TaPOEI* family members [25,26,27,28]. These target genes primarily belong to clades 4 and 5 of the phylogenetic tree (Figure 2). Tae-miR9676-5p and tae-miR9780 are predicted to each regulate six target genes; the candidate genes targeted by tae-miR9676-5p are predominantly located on chromosome 4 and belong to clade 6, while those putatively targeted by tae-miR9780 are mainly situated on chromosome 3 and belong to clade 3. Additionally, tae-miR9677a, which has been implicated in male fertility across various male-sterile wheat lines, is predicted to regulate three target genes: *TaPOEI 27-B*, *TaPOEI 27-D*, and *TaPOEI 30-D*. These findings suggest a complex regulatory network in which specific miRNAs modulate the expression of *TaPOEI* genes, potentially influencing reproductive development and stress responses in wheat. Functional validation of these miRNA-target interactions will be necessary to elucidate their precise roles in wheat biology.

### 2.4. Tissue-Specific and Anther-Preferential Expression of TaPOEI Genes: Implications for Functional Roles in Reproductive Development

To elucidate the expression patterns of *TaPOEI* gene family members across various wheat tissues, transcriptome data were retrieved from the WheatOmics1.0 database. A heatmap illustrating the expression levels of *TaPOEI* genes was generated using TBtools software (version 2.305), based on samples collected from roots, stems, leaves, spikes, and grains (Figure S4). Analysis of these transcriptome data revealed that several *TaPOEI* genes are highly expressed in spike tissues and/or leaves. Specifically, genes such as *TaPOEI 3-A*, *TaPOEI 3-B*, *TaPOEI 3-D*, *TaPOEI 15b-A.1*, *TaPOEI 15b-A.3*, *TaPOEI 15b-A.4*, *TaPOEI 15b-A.5*, *TaPOEI 15b-B.2*, *TaPOEI 15b-D*, *TaPOEI 15d-A.2*, *TaPOEI 15d-B*, *TaPOEI 15e-A.1*, *TaPOEI 15e-A.2*, *TaPOEI 15e-B.1*, *TaPOEI 16-A*, *TaPOEI 16-B*, *TaPOEI 16-D*, *TaPOEI 20-A*, *TaPOEI 20-B*, *TaPOEI 20-D*, *TaPOEI 21-A*, *TaPOEI 21-B*, *TaPOEI 21-D*, *TaPOEI 25-A*, *TaPOEI 25-B*, and *TaPOEI 25-D* were highly expressed in spikes and leaves. Additionally, genes such as *TaPOEI 1-B*, *TaPOEI 15a-U.3*, *TaPOEI 16-A*, *TaPOEI 16-B*, *TaPOEI 16-D*, *TaPOEI 20-A*, *TaPOEI 20-B*, *TaPOEI 20-D*, *TaPOEI 21-A*, *TaPOEI 21-B*, *TaPOEI 21-D*, *TaPOEI 25-A*, *TaPOEI 25-B*, and *TaPOEI 25-D* were specifically expressed in spike tissues.

**Figure 3:**
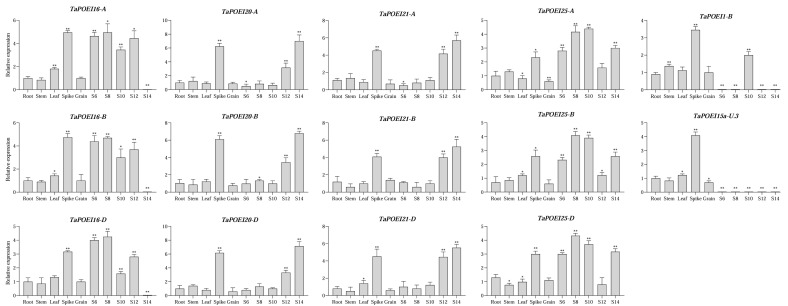
RT-qPCR analysis of *TaPOEI* genes in various tissues. The tissues analyzed were roots, stems, leaves, spikes, grains, and anthers at the central callose (S6), tetrad (S8), vacuolate microspore (S10), 3-nucleate pollen (S12), and dehiscence (S14) stages. The relative expression levels were normalized to 1 in roots. *TaACTIN* was used as a reference gene. Error bars indicate standard deviations based on three biological replicates. Gene expression levels are shown relative to root, and other tissues were normalized accordingly. Statistical significance was determined using Welch’s *t*-test (* for *p* < 0.05 and ** for *p* < 0.01).

To validate these transcriptome data, we performed an RT-qPCR analysis on selected pollen-preferential *TaPOEI* genes. The expression patterns obtained from RT-qPCR were consistent with those observed in the transcriptome datasets (Figure 3). Further analysis revealed that *TaPOEI 20-A*, *TaPOEI 20-B*, *TaPOEI 20-D*, *TaPOEI 21-A*, *TaPOEI 21-B*, and *TaPOEI 21-D* exhibited high expression in anther tissues during the flowering stage. Additionally, *TaPOEI 16-A*, *TaPOEI 16-B*, *TaPOEI 16-D*, *TaPOEI 25-A*, *TaPOEI 25-B*, and *TaPOEI 25-D* were specifically expressed during the earlier stages of anther development (Stages S6 to S12). These findings underscore the tissue- and developmental stage-specific roles of *TaPOEI* genes, particularly in anther tissues, and highlight the need for detailed functional analysis of these genes preferentially expressed in anthers.

### 2.5. Expression Patterns of TaPOEI Genes Under Drought, Salinity, Cold, and Heat Stress: Insights into Stress-Responsive Mechanisms

To explore the expression profiles of the *TaPOEI* gene family under various abiotic stress conditions, we analyzed transcriptome data from publicly available databases and created a heatmap using TBtools software. The expression of *TaPOEI* genes in response to drought, salinity, cold, and heat stresses was examined (Figure S5). Under drought stress, most *TaPOEI* genes were downregulated, with only a few genes being upregulated. Notably, *TaPOEI 19-B*, *TaPOEI 24-A*, *TaPOEI 24-B*, *TaPOEI 24-D*, and *TaPOEI 30-D* displayed significant expression differences between drought-tolerant and -sensitive wheat cultivars. In response to salinity stress, approximately half of the *TaPOEI* genes exhibited very low expression levels. Regardless of the salinity tolerance of the wheat cultivar, these genes were generally downregulated under salinity stress. Cold stress induced a significant upregulation of *TaPOEI 20-A*, while *TaPOEI 24-B* and *TaPOEI 24-D* were significantly downregulated. During heat stress, around one-fifth of all *TaPOEI* genes exhibited substantial changes in their expression levels. Specifically, *TaPOEI 16-A*, *TaPOEI 20-A*, *TaPOEI 20-B*, *TaPOEI 20-D*, *TaPOEI 21-A*, *TaPOEI 21-B*, *TaPOEI 21-D*, *TaPOEI 24-A*, *TaPOEI 24-B*, and *TaPOEI 24-D* were significantly downregulated in heat-sensitive materials. Conversely, *TaPOEI 15a-U.1*, *TaPOEI 15a-U.4*, *TaPOEI 15a-U.5*, *TaPOEI 15a-U.6*, *and TaPOEI 15e-U.1* were upregulated in heat-sensitive materials but downregulated in heat-tolerant ones.

**Figure 4:**
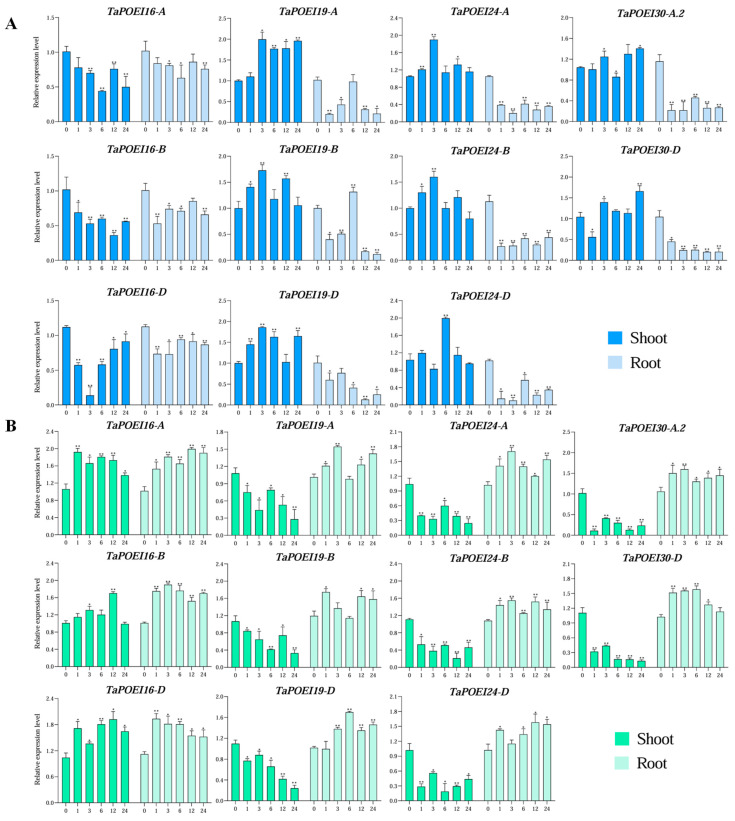

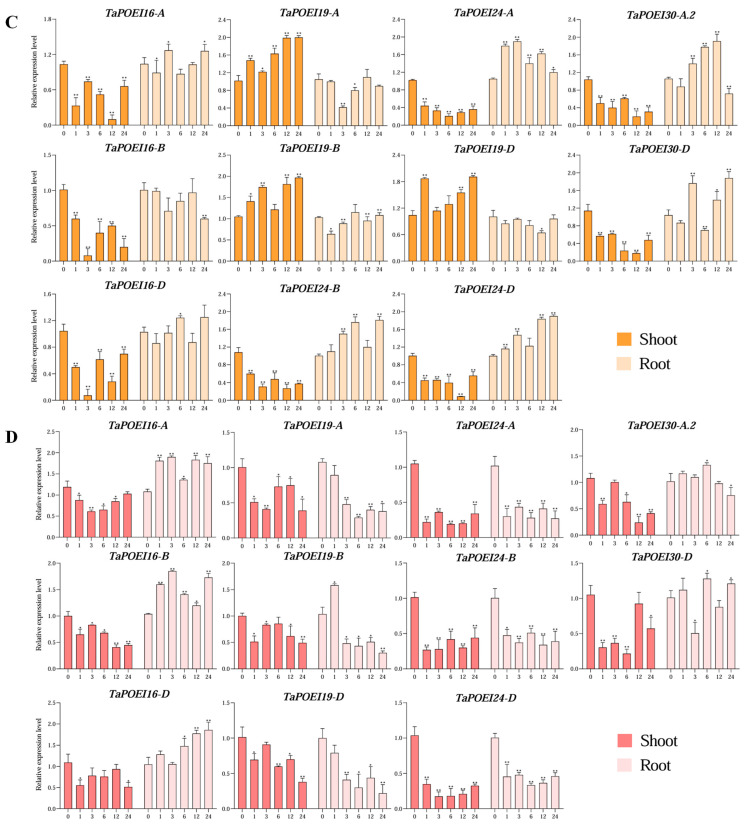
RT-qPCR analysis of 11 *TaPOEI* genes in response to various abiotic stresses. (**A**) Drought (20% [*w*/*v*] PEG6000), (**B**) salinity (200 mM NaCl), (**C**) cold (4 °C), and (**D**) heat (40 °C). The *y*-axis represents relative expression levels, and the *x*-axis represents the time point (0, 1, 3, 6, 12, or 24 h into treatment). *TaACTIN* was used as a reference gene. Error bars indicate standard deviations based on three biological replicates. Gene expression levels are shown relative to the control (0 h), and other treatments were normalized accordingly. Statistical significance was determined using Welch’s *t*-test (* for *p* < 0.05 and ** for *p* < 0.01).

To validate these findings, we conducted RT-qPCR on selected genes potentially involved in multiple stress responses: *TaPOEI 16-A*, *TaPOEI 16-B*, *TaPOEI 16-D*, *TaPOEI 19-A*, *TaPOEI 19-B*, *TaPOEI 19-D*, *TaPOEI 24-A*, *TaPOEI 24-B*, *TaPOEI 24-D*, *TaPOEI 30-A.2*, and *TaPOEI 30-D*. We assessed their expression levels in both shoot and root tissues of seedlings from the wheat cultivar Chinese Spring (Figure 4). The results largely corroborated the transcriptome data, with expression patterns in shoot tissues mirroring those observed in the downloaded datasets. In root tissues, *TaPOEI 19-A*, *TaPOEI 19-B*, and *TaPOEI 19-D* were significantly repressed under drought, cold, and heat stress conditions, while their expression was notably suppressed in shoot tissues under salinity stress. *TaPOEI 16-A*, *TaPOEI 16-B*, and *TaPOEI 16-D* showed no significant changes in expression under drought or salinity stress conditions, but they were significantly downregulated in shoot tissues under cold stress and upregulated in root tissues under heat stress. *TaPOEI 30-A.2*, and *TaPOEI 30-D* exhibited similar expression patterns under salinity, cold, and heat stress conditions, characterized by their downregulation in shoot tissues and their upregulation in root tissues. Under drought stress, these genes were significantly downregulated in root tissues, with no significant changes in shoot tissues. Similarly, *TaPOEI 24-A*, *TaPOEI 24-B*, and *TaPOEI 24-D* expression levels were downregulated in shoot tissues and upregulated in root tissues under salinity and cold stress conditions, but they were downregulated in both tissues under heat stress.

### 2.6. Expression Dynamics and Functional Insights of Anther-Preferential TaPOEI Genes in TGMS Wheat Lines Under Low-Temperature Conditions

**Figure 5:**
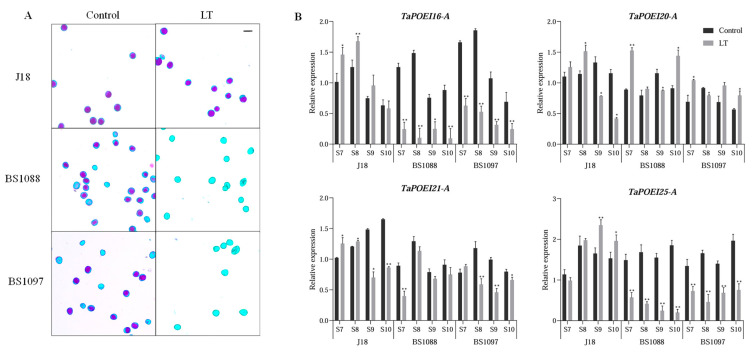
(**A**) Morphological characteristics of pollen grains from TGMS lines (BS1088 and BS1097) and the common wheat cultivar (J18) stained with Alexander stain, observed under moderate temperature (control) conditions promoting fertility and low-temperature (LT) sterile conditions. The control pollen grains are fully developed, exhibit near-complete starch accumulation, stain dark blue, and appear large and plump, and the sterile pollen grains show markedly reduced starch accumulation, appear smaller and shriveled, and fail to stain. Scale bars represent 100 μm. (**B**) Gene expression profiles with anther preference in TGMS lines and the common wheat cultivar under both fertile conditions and low-temperature sterile conditions. *TaACTIN* was used as a reference gene. The black and gray columns represent the moderate temperature (control) fertile conditions and low-temperature (LT) sterile conditions. Error bars indicate standard deviations based on three biological replicates. Gene expression levels are shown relative to the S7-stage anther of the common wheat cultivar (J18). Other developmental stages and cultivars were normalized accordingly. Statistical significance was determined using Welch’s *t*-test (* for *p* < 0.05 and ** for *p* < 0.01).

To elucidate the role of *TaPOEI* genes preferentially expressed in anthers during anther development, we investigated their expression profiles in thermosensitive genic male-sterile (TGMS) wheat lines under low-temperature-induced sterility conditions. Specifically, we used the lines J18, BS1088, and BS1097, grown under control high-temperature fertile conditions or the low-temperature sterile condition, as assessed by Alexander staining of fresh pollen grains [29,30]. Our previous research demonstrated that, under moderate temperatures conducive to fertility, the pollen morphology of thermosensitive genic male-sterile (TGMS) lines closely resembles that of common wheat cultivars. In these conditions, pollen grains are fully developed, exhibit near-complete starch accumulation, stain dark blue, and appear large and plump [29,30,31]. By contrast, under low-temperature conditions that induce sterility, pollen grains from TGMS lines show markedly reduced starch accumulation, appear smaller and shriveled, and fail to stain, indicating developmental abnormalities. In contrast, the pollen grains of the common wheat cultivar maintain normal morphology and staining under both conditions (Figure 5A).

Using RT-qPCR, we measured the expression of these genes in male-sterile and fertile wheat lines across the anther development stages S7 to S10 (Figure 5 and Figure S8). RT-qPCR analysis revealed that *TaPOEI 20-A* and *TaPOEI 21-A* are expressed at similar levels in sterile and fertile lines under both low-temperature sterility and normal-fertility conditions (Figure 5). Similarly, in the common wheat cultivar J18, the expression of *TaPOEI 16-A* and *TaPOEI 25-A* remained stable regardless of the growth conditions. However, in TGMS lines under a low-temperature-induced sterility condition, the expression levels of *TaPOEI 16-A* and *TaPOEI 25-A* were significantly downregulated compared to that seen in normal temperature conditions. This differential expression underscores the potential role of these genes in anther development and fertility, particularly under stress conditions that disrupt normal pollen formation.

## 3. Discussion

The *POEI* genes are crucial for various biological functions that influence plant growth and development, particularly in response to environmental stresses [7,14,17]. In this study, our comprehensive identification and characterization of the *POEI* genes in wheat provide valuable insights into their distribution, evolutionary relationships, structural characteristics, regulatory mechanisms, and expression patterns. Specifically, we identified *TaPOEI 16-A*, an anther-preferential *POEI* gene, whose expression not only responds to various abiotic stresses but also appears to be involved in male sterility, suggesting its potential role in regulating fertility in response to temperature fluctuations.

Our phylogenetic analysis of the reported *POEI* genes (Figure S6) revealed that anther-specific genes expressed during later stages of anther development (*TaPOEI 20-A*, *TaPOEI 20-B*, *TaPOEI 20-D*, *TaPOEI 21-A*, *TaPOEI 21-B*, and *TaPOEI 21-D*) are closely related to *LAT52* and *Zm13*. These findings suggest their potential involvement in pollen tube development [17,22]. Notably, abiotic stress-related genes such as *TaPOEI 24-A*, *TaPOEI 24-B*, and *TaPOEI 24-D* were also closely related to these genes, indicating a potential role for these abiotic stress-responsive genes in pollen development. Furthermore, *TaPOEI 16-A*, *TaPOEI 16-B*, and *TaPOEI 16-D*, which are preferentially expressed in anthers, were found to be phylogenetically distant from the aforementioned genes, suggesting they may serve distinct functions in pollen development.

Interestingly, *OsPOEI 16*, a homolog of *TaPOEI 16-A*, was also found to exhibit anther-preferential expression and respond significantly to both cold and heat stresses, suggesting that these genes might share similar functions through multiple temperature-regulated pathways (Figures S9 and S10). This observation highlights the evolutionary conservation of *POEI* genes across species, with potential roles in regulating temperature-sensitive processes, including male fertility.

MicroRNAs are crucial regulators of gene expression, particularly during reproductive development [25,27]. In this study, we identified tae-miR1124 and tae-miR9677a as potential regulators of multiple *TaPOEI* genes, particularly those associated with anther development and fertility. Additionally, tae-miR531, which has been implicated in drought tolerance, is predicted to target *TaPOEI 25-B* and *TaPOEI 25-D*, further suggesting these genes may contribute to drought stress responses. Notably, tae-miR9657a-3p, which exhibits differential abundance between sterile and fertile conditions, was predicted to target *TaPOEI 16-B* and *TaPOEI 16-D*, implicating it in the regulation of male sterility in wheat. Previous studies have suggested that tae-miR9657 contributes to salt tolerance in wheat by being downregulated in salt-tolerant cultivars, thereby enhancing the expression of its target genes encoding MYB-related transcription factors [32]. These findings indicate that tae-miR9657 is involved not only in abiotic stress responses but also in fertility regulation. In future work, we plan to experimentally validate these regulatory relationships through miRNA overexpression or knockdown approaches, which will help clarify the functional roles of miRNA-mediated control of *TaPOEI* genes in stress adaptation and male fertility. These findings establish a foundation for future genetic and breeding strategies aimed at enhancing wheat’s stress resilience and fertility.

The tissue-specific expression patterns of *TaPOEI* genes, particularly their spike-preferential expression, suggest that these genes may contribute to reproductive development, especially pollen formation and pollen fertility [17]. The expression levels of *TaPOEI* genes from different clades exhibit varying sensitivities to stressors: clade 2 members primarily responded to drought, salinity, and cold stress; those of clade 3 were notably associated with salinity stress responses; and clades 4 to and 6 were more responsive to heat stress (Figure S5). This clade-specific response suggests evolutionary adaptations that enable wheat to cope with diverse environmental stresses through distinct sets of *TaPOEI* genes. In soybean, the overexpression of *GmPO I* enhances drought tolerance, while *AtPRPL1* in Arabidopsis plays a key role in the salinity stress response [17,18]. *GmPO I* was reported to potentially be induced by cold, salt, and drought stress; moreover, the overexpression lines significantly enhanced drought stress. In our study, the wheat genes *TaPOEI 43-A.1*, *TaPOEI 43-B.1*, and *TaPOEI 43-D.1*, which share high sequence identity with these proteins (Figure 1 and Figure S6), showed significant downregulation under drought but upregulation under salinity stress (Figure S7). These results suggest a specific role for these wheat genes in drought response, potentially reflecting a functional alignment closer to soybean genes rather than to Arabidopsis. This divergence in functional adaptation, even among homologous genes, highlights how evolutionary pressures may drive specialization for distinct environmental stress responses across species. The observed downregulation of most *TaPOEI* genes under drought stress, alongside their selective upregulation in drought-tolerant wheat lines, suggests that they may modulate stress tolerance mechanisms. Similarly, contrasting expression patterns under salinity and heat-stress conditions between sensitive and resistant lines suggests that these genes may serve as biomarkers for breeding stress-tolerant wheat cultivars.

This study explored the expression profiles of anther-preferential *TaPOEI* genes in TGMS wheat under low-temperature-induced sterility conditions. In TGMS lines under low-temperature conditions, *TaPOEI 16-A* and *TaPOEI 25-A* showed pronounced downregulation. This downregulation coincided with abnormal pollen morphology, including shriveled and starch-deficient grains, compared to fertile controls (Figure 5A). In contrast, the expression of *TaPOEI 20-A* and *TaPOEI 21-A* remained relatively stable across both conditions, suggesting that these genes may play more general roles in anther or pollen development, rather than contributing to temperature-sensitive male sterility. Notably, the expression of genes such as *TaPOEI 16-A* and *TaPOEI 25-A* was downregulated in these sterile lines, while their expression remained stable in fertile lines. This differential expression suggests a potential role for these genes in regulating fertility in response to temperature fluctuations. *TaPOEI 20-A* and *TaPOEI 21-A* did not show significant expression differences between sterile and fertile conditions, suggesting that they may play a broader role in anther development rather than directly contributing to sterility. This observation provides a new direction for targeted research into the molecular mechanisms governing TGMS in wheat, particularly the manipulation of specific *TaPOEI* genes to control fertility, which could enhance hybrid breeding strategies.

Bioinformatics analysis conducted in this study identified *TaPOEI 16-A* and *TaPOEI 25-A* as potentially involved in fertility regulation in wheat TGMS lines. Particularly, *TaPOEI 16-A* expression was responsive to temperature changes, suggesting its role in the induction of sterility through temperature-sensitive regulation during anther development. Previous research identified a thermo-photoperiod-sensitive male-sterile line, 337S, the first wheat line known to be sensitive to both low-temperature/short-daylength and high-temperature/long-daylength conditions [33]. The significant drop in *TaPOEI 16-A* expression in low-temperature sterile lines supports its potential involvement in low-temperature-induced male sterility. Furthermore, expression analysis under various abiotic stresses revealed that *TaPOEI 16-A* expression also responded to high-temperature stress, indicating that it may influence wheat fertility through multiple temperature-regulated pathways. It is hypothesized that *TaPOEI 16-A* expression is regulated by temperature, particularly in sterile TGMS lines. During the critical stages of anther development, a drop in temperature may severely repress *TaPOEI 16-A* expression, thereby disrupting normal anther development, leading to pollen abortion, and ultimately reducing wheat fertility and yield. Future studies involving gene editing and overexpression experiments are planned to explore the function of *TaPOEI 16-A* and elucidate its role in fertility regulation pathways.

This comprehensive analysis of the *TaPOEI* gene family provides a solid foundation for functional studies and genetic improvement efforts. The tissue-specific and stress-responsive expression patterns of the *TaPOEI* gene family members, along with their possible roles in anther development, underscore their importance in reproductive biology and stress acclimation. The findings of this study lay the groundwork for the functional characterization of these genes, which could ultimately lead to the development of wheat cultivars with enhanced fertility and resilience to environmental stresses. Integrating genomic, phylogenetic, and expression data presents a holistic view of the *TaPOEI* genes, paving the way for innovative approaches to boost wheat productivity and resilience in the face of global climate challenges.

## 4. Materials and Methods

### 4.1. Identification and Characteristics of POEI Genes in Wheat

We systematically identified and characterized the *POEI* gene family within the wheat genome using Pollen Ole e I protein sequences from Arabidopsis and rice as query sequences. We conducted a genome-wide search using BLASTP (https://blast.ncbi.nlm.nih.gov/Blast.cgi (accessed on 6 January 2024)) and HMMER (https://www.ebi.ac.uk/Tools/hmmer/home (accessed on 6 January 2024)) tools, focusing on the identification of putative *POEI* genes (IWGSC RefSeq v2.1). The resulting sequences were validated for the presence of the Pollen Protein Ole e1 domain using the Pfam database and the Conserved Domain Database at NCBI. Physicochemical properties of the identified proteins, such as amino acid composition, molecular weight, and isoelectric point (pI), were determined using the ProtParam tool on the ExPASy server (https://www.expasy.org/ (accessed on 13 May 2024)) [29,30].

### 4.2. Chromosomal Location and Phylogenetic Relationships of POEI Genes

The chromosomal distribution of the identified *TaPOEI* genes was visualized using the MapGene2Chromosome tool (http://mg2c.iask.in/mg2c_v2.0/ (accessed on 20 January 2024)), enabling the precise mapping of these genes across the wheat genome. To explore the evolutionary relationships of POEI proteins, homologs in Arabidopsis were identified through the Ensembl Plants database (http://plants.ensembl.org/hmmer/index.html (accessed on 20 January 2024)). A phylogenetic tree was reconstructed using MEGA-X software, incorporating wheat and Arabidopsis protein sequences, utilizing the maximum likelihood method with 1000 bootstrap replicates to ensure robust phylogenetic clustering.

### 4.3. Analysis of Gene Structures, Conserved Motifs, Promoter Elements, and Expression Analysis

Gene structure visualization was performed using the Gene Structure Display Server (http://gsds.cbi.pku.edu.cn/ (accessed on 13 May 2024)) to gain insights into exon–intron organization within the *TaPOEI* gene family. Conserved motifs in the encoded proteins were identified using the MEME Suite (http://meme-suite.org (accessed on 13 May 2024)), with a maximum of 10 motifs allowed, and the results were visualized through TBtools (version 2.305). To investigate the regulatory mechanisms of *TaPOEI* gene expression, the promoter regions (2 kb upstream of the coding sequences) of *TaPOEI* genes were analyzed for cis-regulatory elements using the PlantCARE database (http://bioinformatics.psb.ugent.be/webtools/plantcare/html/ (accessed on 13 May 2024)), with subsequent visualization facilitated by TBtools. Domain conservation among TaPOEI proteins was assessed through sequence alignment using MEGA 10 software.

Expression data from RNA-seq and microarray studies of the wheat cultivar ‘Chinese Spring’ (CS) were retrieved from the WheatOmics database (http://wheatomics.sdau.edu.cn (accessed on 3 July 2024)). Expression data from Arabidopsis, wheat, rice, and soybean were obtained from public repositories: https://plantrnadb.com, https://evorepro.sbs.ntu.edu.sg, and https://plantrnadb.com (accessed on 3 July 2024) [34,35]. To assess tissue-specific expression patterns under various abiotic stress conditions (drought, salinity, cold, and heat), transcript per million values were extracted and visualized as heatmaps using TBtools software [29,30].

### 4.4. Prediction of microRNA Targets

MicroRNA (miRNA) target sites in the *TaPOEI* gene family were predicted using the psRNATarget platform (https://www.zhaolab.org/psRNATarget/ (accessed on 13 May 2024)), based on sequence complementarity with known wheat miRNAs [26]. Additionally, small RNA targets were predicted using the psRobot tool (https://www.zhaolab.org/psRNATarget/ (accessed on 13 May 2024)), and the resulting regulatory networks were then constructed and visualized using Cytoscape v3.8.2 software.

### 4.5. Plant Materials and Stress Treatments

To determine the tissue-specific expression patterns of *TaPOEI* genes, samples were collected from various tissues of the wheat cultivar CS, namely, roots and shoots at the seedling stage, leaves at the tillering stage, spikes and anthers at the heading stage, and grains at the milking stage [30]. Additionally, anthers at the central callose (S6), tetrad (S8), vacuolate microspore (S10), 3-nucleate pollen (S12), and dehiscence (S14) stages were collected from CS [36,37,38]. For abiotic stress treatments, seeds were first germinated on water-soaked filter paper at 23 °C under a 16 h light/8 h dark photoperiod. After germination, seedlings were transferred to mesh grids and kept at 28 °C under the same photoperiod. Two-week-old seedlings were subjected to drought (20% [*w*/*v*] polyethylene glycol 6000 [PEG6000]), salinity (200 mM NaCl), cold (4 °C), or heat (40 °C) stress for varied durations (0, 1, 3, 6, 12, or 24 h) [29,30]. To study fertility and sterility, the TGMS lines BS1088 and BS1097 were cultivated under temperature conditions conducive to either fertility or sterility, with the wheat cultivar ‘Jing18’ (J18) used as a fertility control. Beijing, characterized by moderate temperatures during anther development, served as the fertile environment, while Nanyang in Henan province, with its low temperatures during anther development, provided the sterile environment [29,30,31]. Anthers were collected at key stages of male reproductive development—meiotic (S7), tetrad (S8), young microspore (S9), and vacuolate microspore (S10)—and immediately frozen in liquid nitrogen for further analysis [29,31,36,37].

### 4.6. RNA Isolation and Reverse-Transcription Quantitative PCR (RT-qPCR) Analysis

Samples from seedlings exposed to abiotic stress and anthers from plants grown under fertile and sterile conditions were collected, flash-frozen in liquid nitrogen, and stored at −80 °C until RNA extraction. Total RNA was isolated using TRIzol reagent (Invitrogen, Waltham, MA, USA) following the manufacturer’s instructions. Complementary DNA (cDNA) synthesis was performed using a Primer Script RT reagent kit with gDNA Eraser (TaKaRa, Kusatsu, Japan). Quantitative PCR (qPCR) was conducted using ChamQ SYBR qPCR Master Mix (Vazyme, Nanjing, China) on a Bio-Rad CFX™ real-time PCR detection system. Primers were designed using Primer Premier 5.0 software, with *TaACTIN* (Gene ID: LOC542814) employed as the internal reference gene (Table S2). Relative expression levels of *TaPOEI* genes were calculated using the 2^−ΔΔCt^ method [29,30].

## 5. Conclusions

The *TaPOEI* gene family plays a critical role in reproductive development and stress responses, particularly in relation to anther-specific expression and fertility regulation. The identification of *TaPOEI 16-A*, which responds to temperature fluctuations and other abiotic stresses, highlights its potential involvement in temperature-induced male sterility. This study provides a foundational understanding that could inform future genetic and breeding strategies aimed at enhancing wheat fertility and stress resilience.

## Data Availability

The original contributions presented in this study are included in the article/Supplementary Materials. Further inquiries can be directed to the corresponding author(s).

## Supplementary Materials

The following supporting information can be downloaded at: https://www.mdpi.com/article/10.3390/ijms26167707/s1.
