# Supplementary material for: Analysis of Wheat Pollen Ole E I Proteins Reveals Potential Roles in Fertility and Stress Adaptation
Source: Int J Mol Sci. 2025 Aug 9;26(16):7707. doi: 10.3390/ijms26167707 (PMC12386451; doi:10.3390/ijms26167707)
Supplement: Supplementary file 1 [file ijms-26-07707-s001.zip › ijms-3749976-supplementary.pdf]

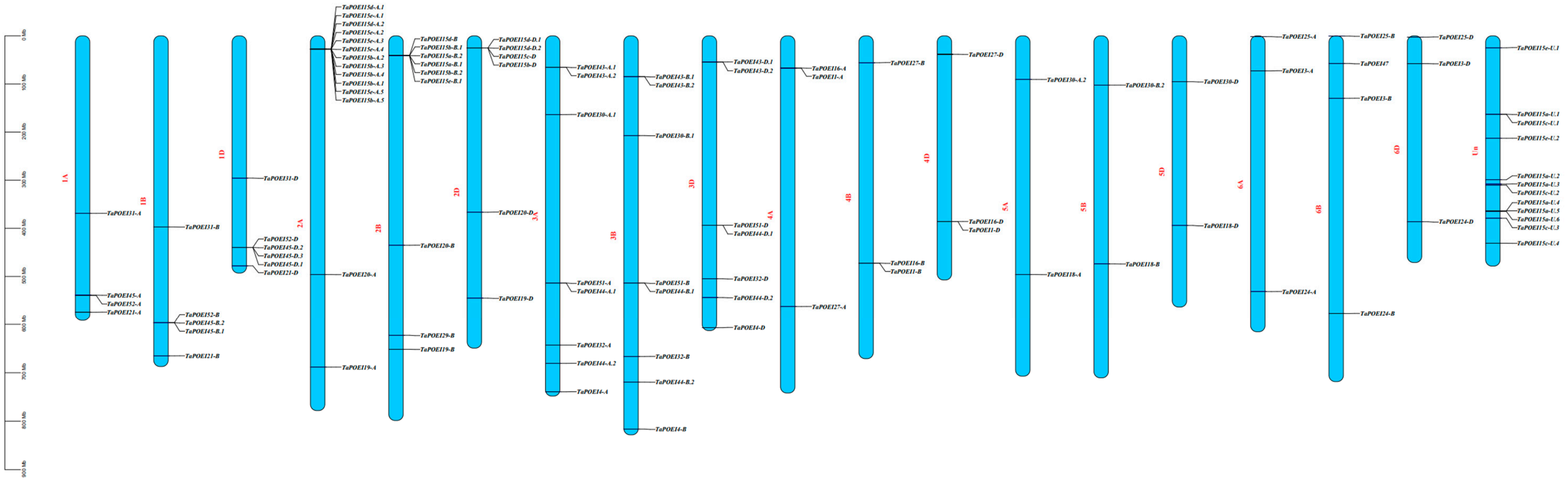

Figure S1. Chromosomal distribution of the identified *TaPOEI* genes across the wheat genome. Chromosome numbers are listed above the chromosomes, and chromosome sizes are shown on the left side of the figure in megabases (Mb).

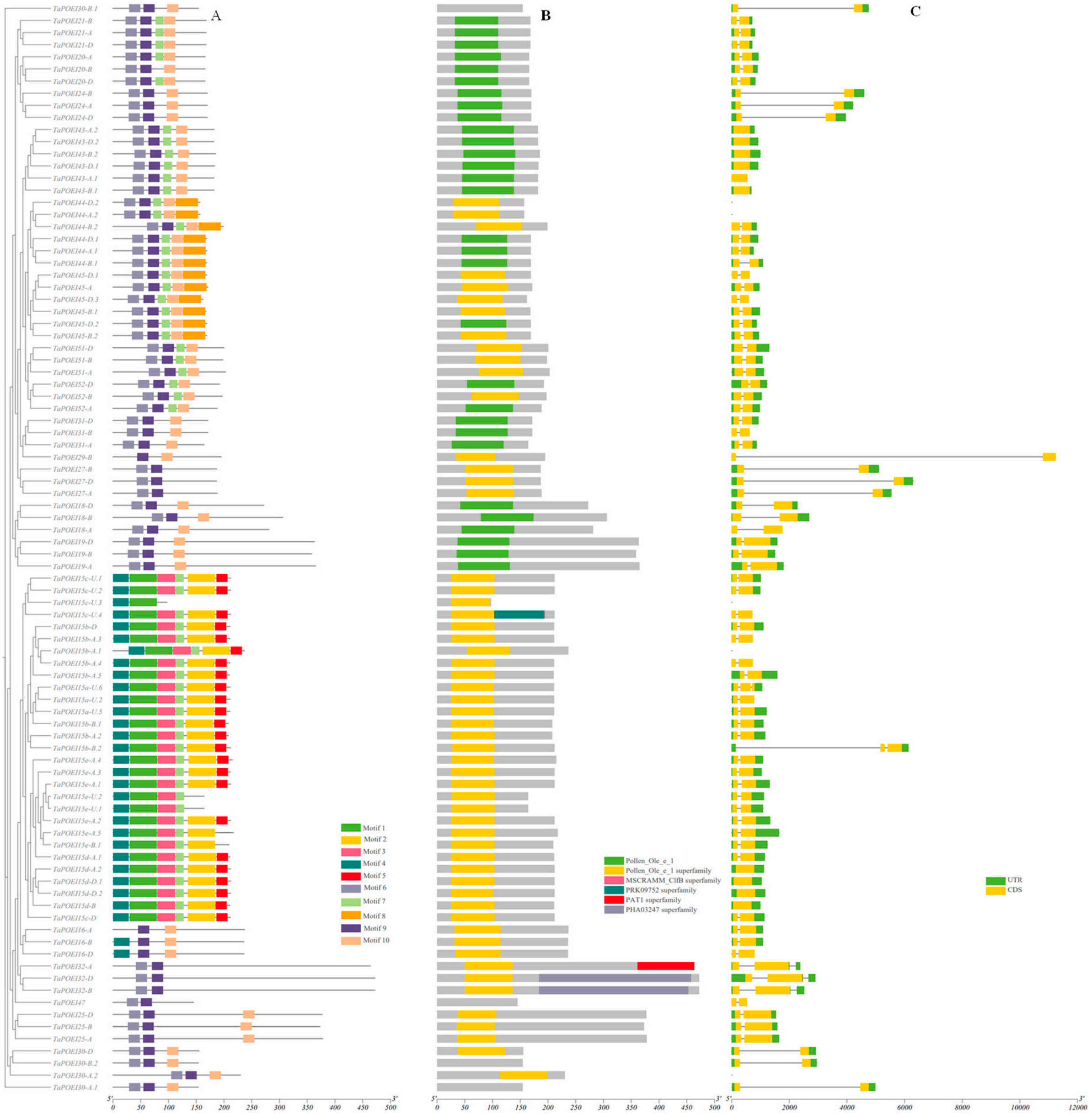

A
B
C
Motif 1
Motif 2
Motif 3
Motif 4
Motif 5
Motif 6
Motif 7
Motif 8
Motif 9
Motif 10
Pollen_Ole_e_1
Pollen_Ole_e_1 superfamily
MSCRAMM_ClfB superfamily
PRK09752 superfamily
PAT1 superfamily
PHA03247 superfamily
UTR
CDS

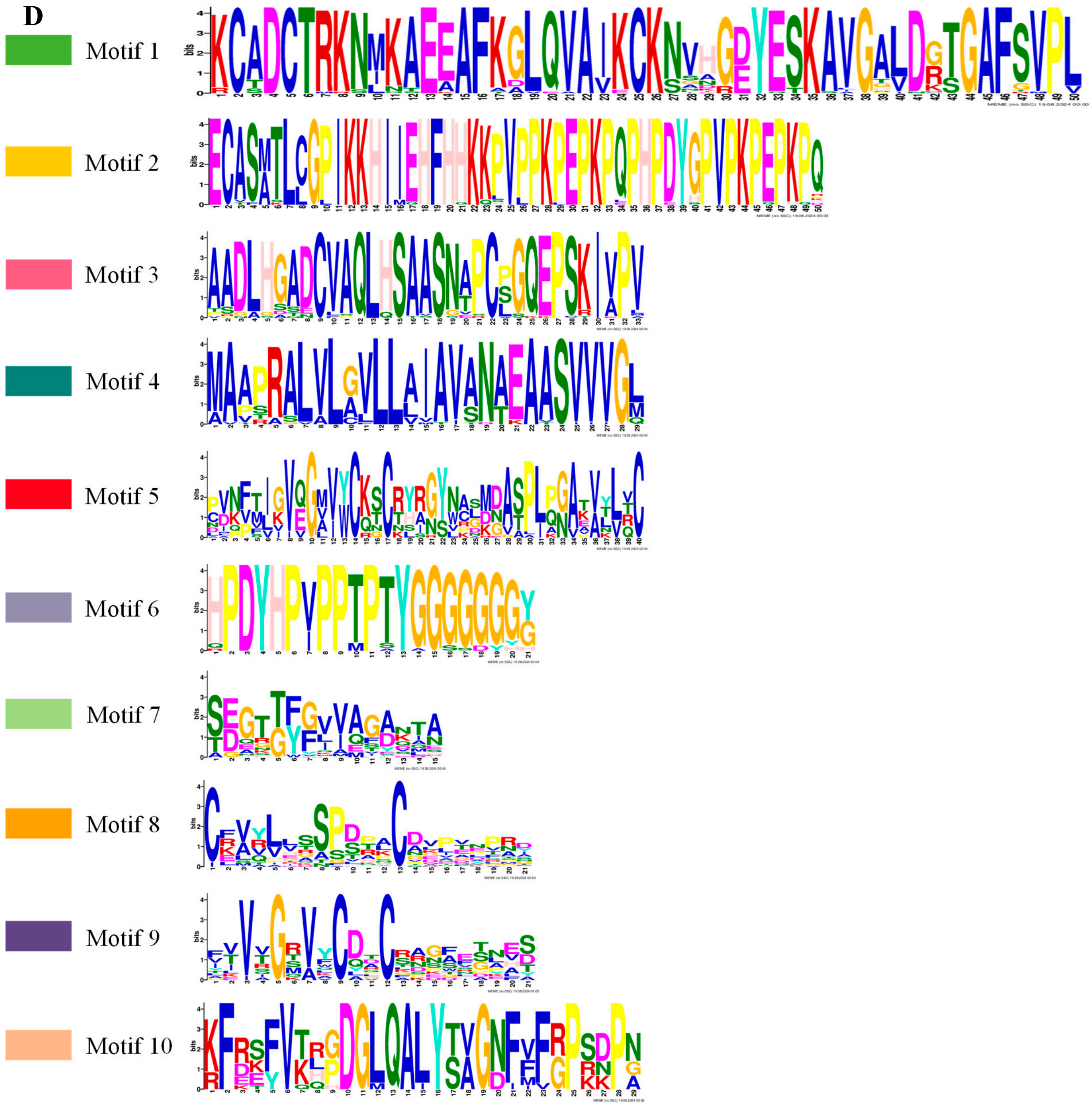

Figure S2. Phylogenetic relationships and analysis of conserved motifs (A), gene structures(B), conserved domain(C) and sequence logo of discovered motifs (D)of *POEI* gene family members in common wheat.

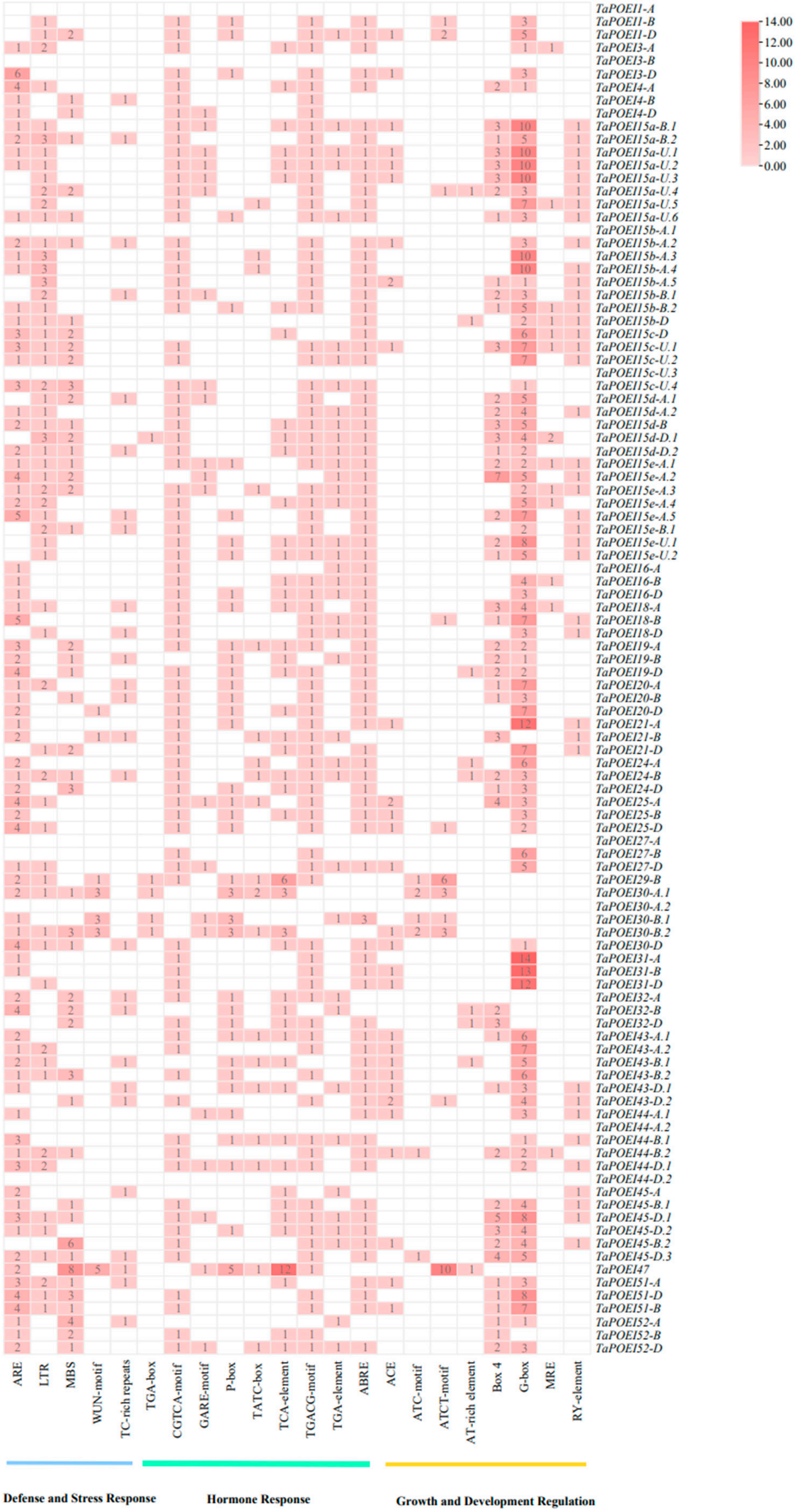

Figure S3. Statistical analysis is of cis-acting regulatory elements of *TaPOEIs* promoters. The color and number of grids represent the number of cis-acting elements in the corresponding *TaPOEIs*.

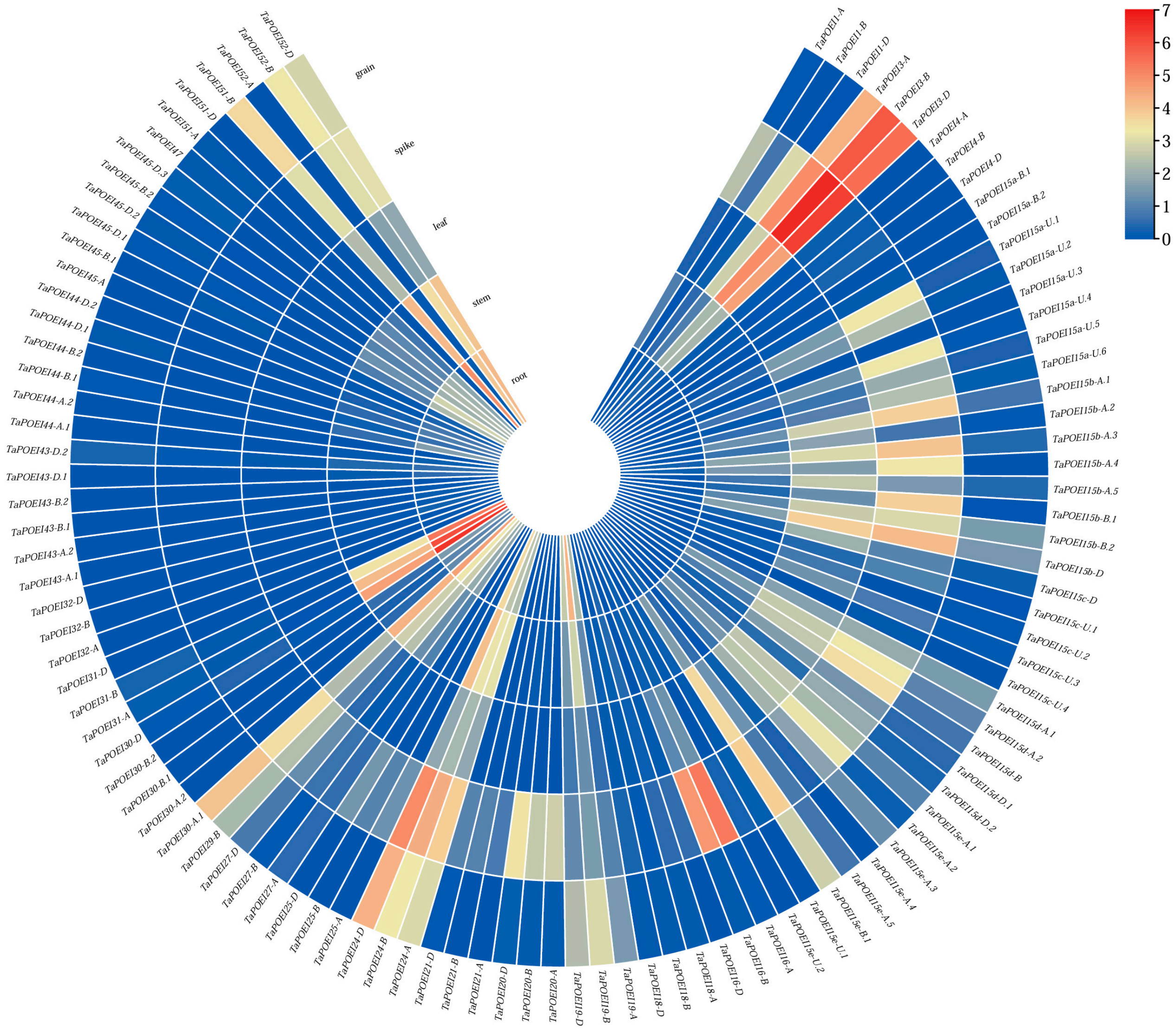

Figure S4. The expression profiles of the *TaPOEIs* involved in 5 tissues (grain, spike, leaf, stem, root) at different growth stages. The heatmap was generated using log_2(TPM) values, with the color scale indicating gene expression levels.

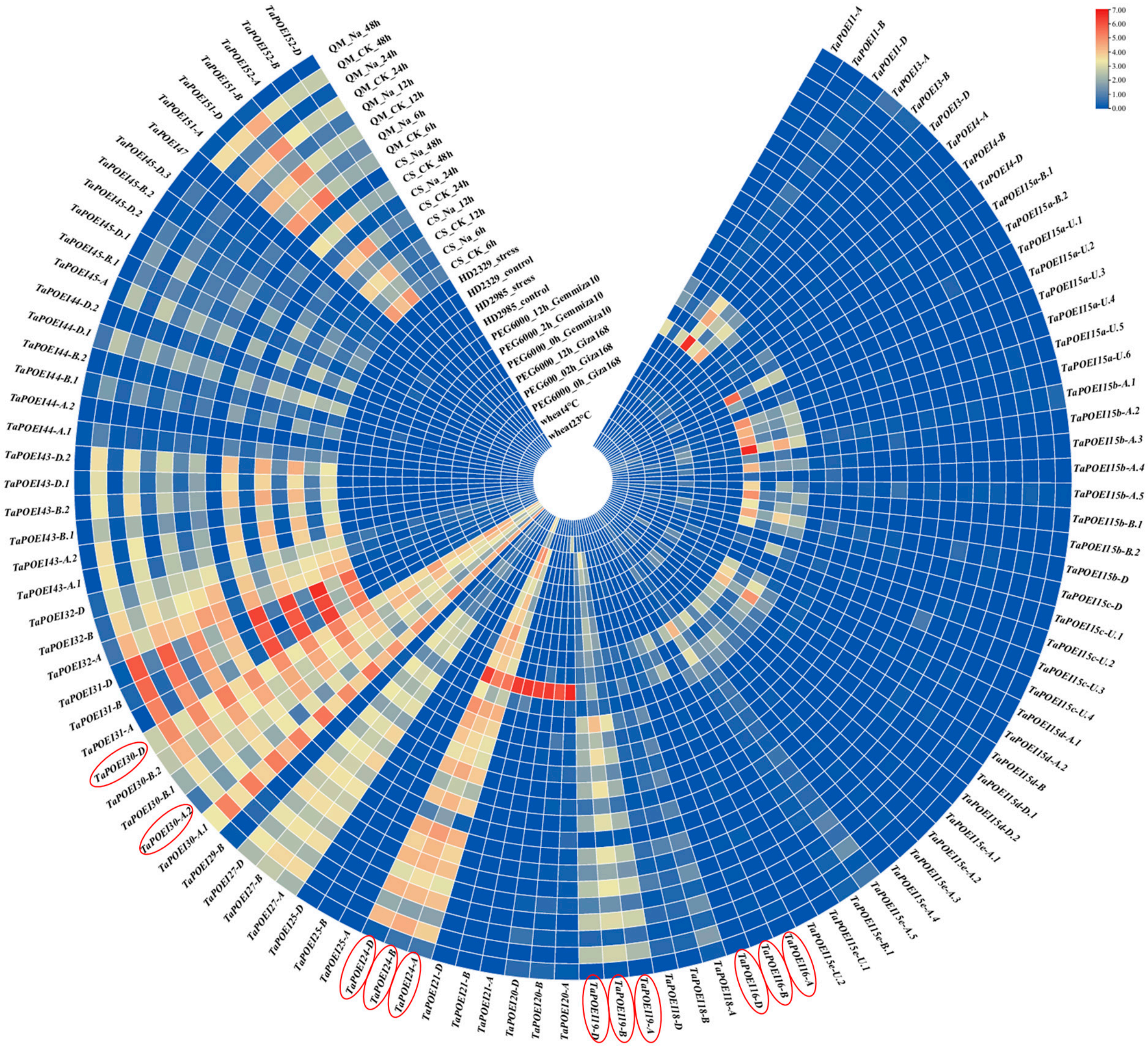

Figure S5. Expression profiles of *TaPOEI* genes in wheat under various abiotic stress conditions. This heatmap presents the expression profiles of *TaPOEI* genes in response to multiple abiotic stresses, including drought, salt, cold, and heat treatments, in different wheat genotypes. Gene expression levels, represented as log_2(TPM) values, are indicated by a color scale, with warmer colors denoting higher expression and cooler colors indicating lower expression. Drought stress: Expression data for Giza168 and Gemmiza10 genotypes under polyethylene glycol (PEG6000)-induced drought stress were recorded at 0, 2, and 12 hours (PEG6000_0h_Giza168, PEG6000_2h_Giza168, PEG6000_12h_Giza168, PEG6000_0h_Gemmiza10, PEG6000_2h_Gemmiza10, and PEG6000_12h_Gemmiza10). Salt stress: The Chinese Spring (CS) and QM genotypes were subjected to salinity treatments, with samples collected at 6, 12, 24, and 48 hours. Control (CK) and salt-treated (Na) conditions are denoted for each time point (CS_CK_6h, CS_Na_6h, CS_CK_12h, CS_Na_12h, CS_CK_24h, CS_Na_24h, CS_CK_48h, CS_Na_48h, QM_CK_6h, QM_Na_6h, QM_CK_12h, QM_Na_12h, QM_CK_24h, QM_Na_24h, QM_CK_48h, QM_Na_48h). Cold stress: Wheat plants were exposed to 4°C, with comparisons drawn to control plants maintained at 23°C (wheat_23°C and wheat_4°C). Heat stress: HD2985 and HD2329 genotypes were analyzed under control and high-temperature conditions (HD2985_control, HD2985_stress, HD2329_control, HD2329_stress). The *TaPOEI* genes selected for further validation of expression levels by RT-qPCR are marked with red circles.

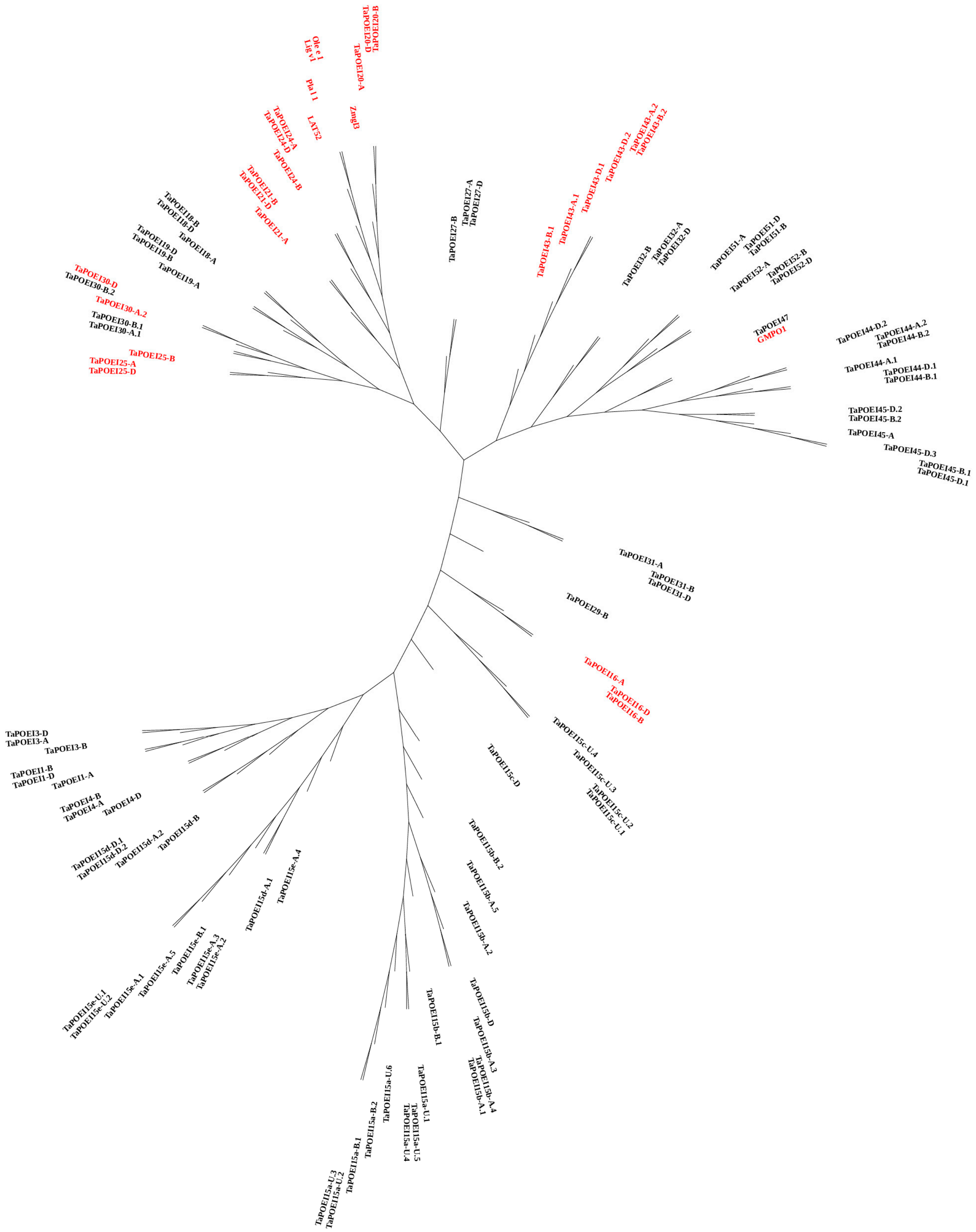

Figure S6. Phylogenetic analysis of the reported POEI proteins and TaPOEI proteins. A maximum-likelihood phylogenetic tree was constructed using MEGA-X software. The POEI proteins reported in other studies, as well as the key proteins focused on in this study, are labeled in red.

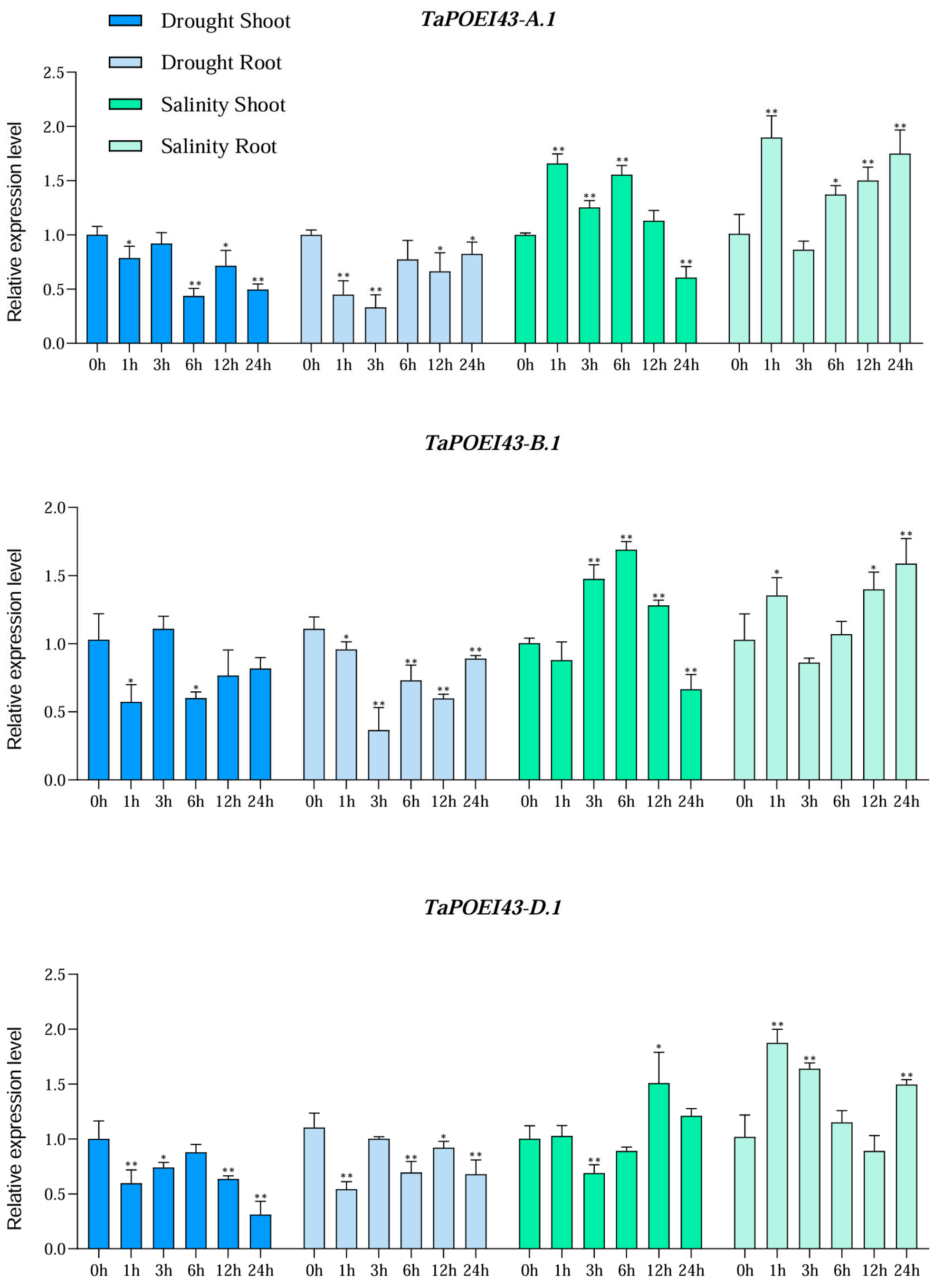

Figure S7. Temporal expression profiles of *TaPOEI43-A.1, TaPOEI43-B.1,* and *TaPOEI43-D.1* genes under drought and salinity stress conditions in wheat. RT-qPCR analysis of *TaPOEI43-A.1, TaPOEI43-B.1,* and *TaPOEI43-D.1* was conducted to assess gene expression dynamics in wheat shoot and root tissues subjected to drought stress (20% [w/v] PEG6000) and salinity stress (200 mM NaCl). Relative expression levels are plotted on the y-axis, normalized to the control condition, with time points at 0, 1, 3, 6, 12, and 24 hours shown on the x-axis. *TaACTIN* was used as a reference gene. Error bars indicate standard deviations based on three biological replicates. Gene expression levels are shown relative to the control (0 h), and other treatments were normalized accordingly. Statistical significance was determined using Welch's *t*-test (* for $p<0.05$ and ** for $p<0.01$).

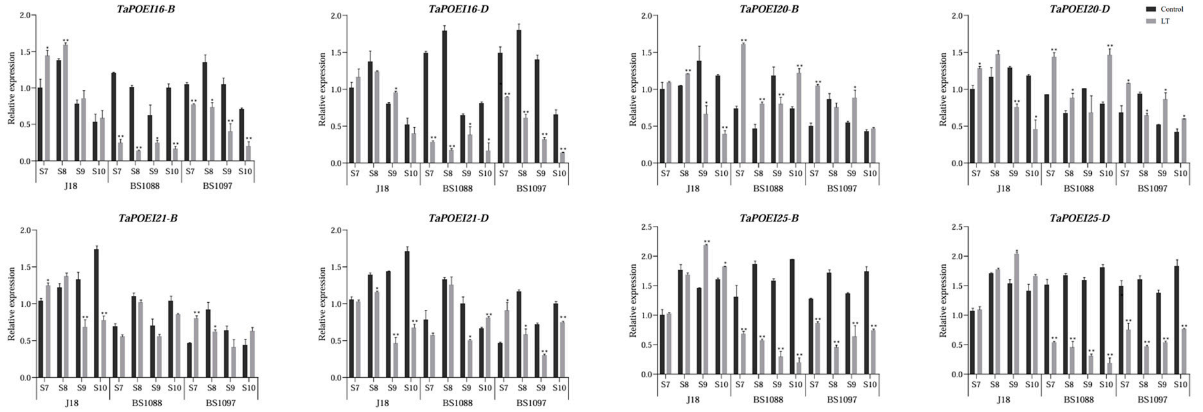

Figure S8. Gene expression profiles with anther-preference in TGMS lines and the common wheat cultivar under both fertile conditions and low-temperature sterile conditions. *TaACTIN* was used as a reference gene. The black and gray columns represent the moderate temperature (Control) fertile conditions and low-temperature (LT) sterile conditions. Error bars indicate standard deviations based on three biological replicates. Gene expression levels are shown relative to the S7-stage anther of the common wheat cultivar (J18). Other developmental stages and cultivars were normalized accordingly. Statistical significance was determined using Welch's *t*-test (* for $p<0.05$ and ** for $p<0.01$).

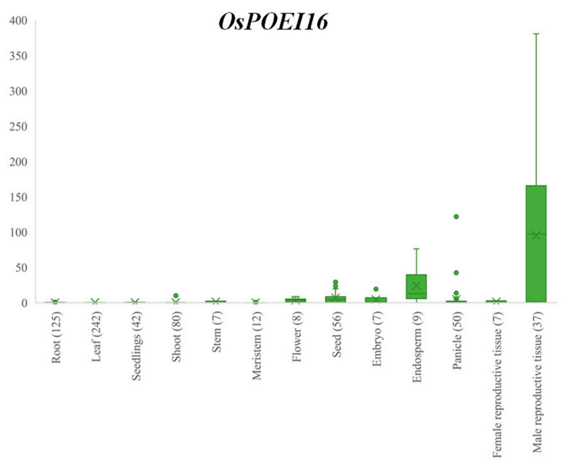

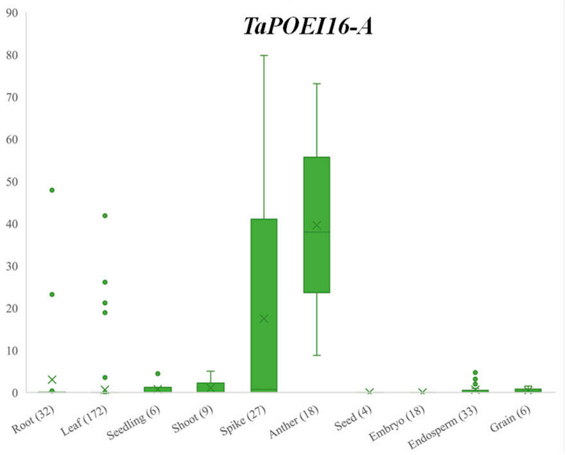

Figure S9. Expression profiles *OsPOEI16* and its homolog gene *TaPOEI16-A* among different tissues according to the Plant Public RNA-seq Database.

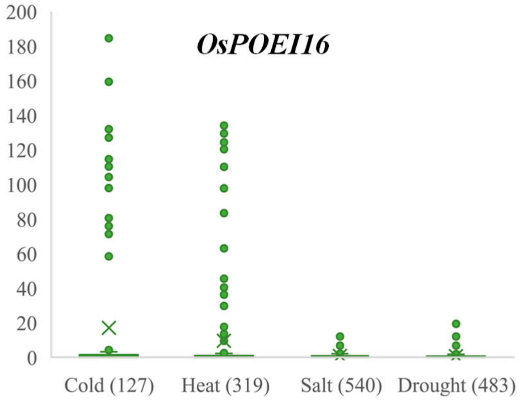

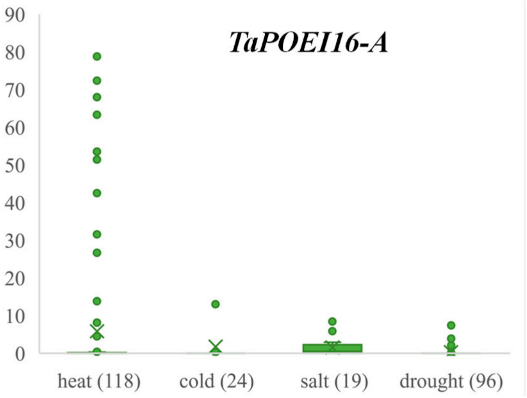

Figure S10. Expression profiles *OsPOEI16* and its homolog gene *TaPOEI16-A* among different abiotic stresses according to the Plant Public RNA-seq Database.
